# Early Sexual Debut and HIV Infection among Men Who Have Sex with Men in Shenzhen, China

**DOI:** 10.1155/2016/2987472

**Published:** 2016-11-28

**Authors:** Ruiwei Xu, Wenjie Dai, Guanglu Zhao, Dan Tu, Lin Yang, Feng Wang, Yumao Cai, Lina Lan, Hongzhuan Tan, Aizhong Liu, Tiejian Feng, Atipatsa C. Kaminga

**Affiliations:** ^1^Department of Epidemiology and Health Statistics, Xiangya School of Public Health, Central South University, Hunan 410008, China; ^2^Shenzhen Center for Chronic Disease Control, Guangdong 518021, China; ^3^Graduate School, Shenzhen University, Guangdong 518060, China; ^4^Department of Mathematics, Mzuzu University, Private Bag 201, Mzuzu 2, Malawi

## Abstract

Studies investigating the association between early sexual debut and human immunodeficiency virus (HIV) infection have mainly focused on Africans or females but rarely on men who have sex with men (MSM) in China. This study, therefore, mainly aimed at exploring the association between early sexual debut and HIV infection among MSM in Shenzhen, China. A total of 533 MSM were enrolled in this study using a convenience sampling method. Information about sociodemographic characteristics and risky sexual behaviors was collected. It was found that the prevalence of HIV infection was 24.2% among this study population and 66.4% of the MSM reported having had vaginal sexual intercourse with females. The mean ages at first vaginal sexual intercourse, first anal sexual intercourse, and first sexual intercourse were 21.38, 22.43, and 19.87 years, respectively. Multivariable logistic regression analyses showed that the MSM who experienced early anal sexual debut were more likely to be infected with HIV than those who did not. The results indicate that HIV infection is quite prevalent among MSM in Shenzhen. Early and efficient intervention strategies should be taken, and the MSM experiencing early anal sexual debut should be given special attention.

## 1. Introduction

In China, the number of individuals reported with human immunodeficiency virus (HIV) has alarmingly increased in recent years, especially among men who have sex with men (MSM) [1]. According to a recent meta-analysis involving twenty-five eligible articles, the pooled HIV incidence among MSM in Mainland China was 5.50/100 person-years in 2012–2014, and MSM only accounted for 21.4% of new HIV infections in China in 2013, but this proportion increased to 25.8% in 2014 [2]. The rapid increase of HIV infection among MSM has brought great challenges to the prevention of HIV infections [3].

Age at first anal sexual intercourse among MSM decreased greatly in the past few decades in China. For instance, among the MSM born in 1990–1996, the median age at first anal sexual intercourse was 18 years, which was less than 33 years, the median age at first anal sexual intercourse among the MSM born in 1940–1959 [4]. Corresponding to the decrease in the age at first anal sexual intercourse was an increase in the number of MSM who experienced early sexual debut, defined as having first sexual intercourse (anal or vaginal) at or before the age of 14 [5]. Early sexual debut may be related to many subsequent risky sexual behaviors, such as having more sexual partners, improper or less condom use during sexual intercourse, and drug use [6, 7]. Thus, individuals with early sexual debut may be more susceptible to HIV infection than those without early sexual debut [5].

Studies investigating the association between early sexual debut and HIV infection have mostly focused on Africans or females but rarely on men who have sex with men (MSM) in China [8, 9]. For example, in rural Malawi and in Nairobi, early vaginal sexual debut (VSD) was significantly associated with the increased risk of HIV infection among ever-married women and HIV-seropositive adolescent girls, respectively [10, 11]. Moreover, in Kenya, men who had sexual debut at the age between 16 and 21 were 1.83 times more likely to be infected with HIV, compared with those who never had sex [12]. In China, early anal sexual debut (ASD) was found to be a risk factor for HIV infection among the MSM without commercial sexual behavior [13].

There were cases in some previous study which indicated that certain proportion of MSM may have bisexual behaviors [14]. Thus, among these MSM, it could be reasonable to divide early sexual debut into early VSD and early ASD when investigating the effect of early sexual debut on HIV infection. Currently, it is not clear in Shenzhen in China if early anal or vaginal sexual debut would be associated with HIV infection among MSM. Therefore, this study mainly aimed at exploring the association between early sexual debut, including early ASD and early VSD, and HIV infection among MSM in Shenzhen, China. Also examined was the association between HIV infection status and their sociodemographic variables and risky sexual behaviors. Age at first sexual intercourse and some sociodemographic factors associated with younger age at first sexual intercourse were also examined.

## 2. Materials and Methods

### 2.1. Ethics Statement

This study was approved by the Ethics Committee of Shenzhen Center for Chronic Disease Control. Written informed consent was obtained from all participants.

### 2.2. Participants

This study was conducted between March 2013 and August 2015 in Shenzhen city of China. Site based convenience sampling method was used to recruit participants. The MSM who came to the sexually transmitted disease (STD) clinic of Shenzhen Center for Chronic Disease Control for treatment or consultation every Monday between March 2013 and August 2015 were considered for enrollment in this study. The inclusion criteria for participants were as follows: (1) being biologically male; (2) self-reporting of having had anal sexual intercourse with at least one biological male in the previous year; (3) age ≥ 18 years; and (4) being willing to provide informed consent. Exclusion criteria included MSM with any kind of mental disease, a history of drug use or prostitution, or severe medical illness. In addition, since this study was a virtually two-year survey, in order to avoid recruiting the same individuals, participants were asked whether or not they had participated in this study before. If the answer was yes, they were excluded.

### 2.3. Data Collection

Data were collected by well-qualified investigators who either had worked for Shenzhen Center for Chronic Disease Control or had studied in a medical school. Before data collection, all investigators went through a unified training which was guided by a written investigation manual. After that, they interviewed the participants face-to-face with an anonymous questionnaire to collect data.

### 2.4. Measurements

#### 2.4.1. Sociodemographic Variables

Sociodemographic variables included in this study were present age, ethnicity, marital status, educational level, socioeconomic disconnection (neither being currently employed nor being a student [15]), monthly income, and length of stay in Shenzhen.

#### 2.4.2. Risky Sexual Variables

Risky sexual variables included the experience of vaginal sexual intercourse, age at first sexual intercourse, age at first anal sexual intercourse, age at first vaginal sexual intercourse, fixed homosexual partner, number of anal sexual partners in the past six months, role in anal sexual intercourse (insertive, receptive, or both insertive and receptive), and condom use in sexual intercourse in the past six months.

Early sexual debut in this study was defined as having first sexual intercourse at the age of 14 or less. Similarly, early ASD and early VSD were, respectively, defined as having first anal sexual intercourse and having first vaginal sexual intercourse at the age of 14 or less. Besides, having a fixed homosexual partner was defined as having an anal sexual relationship with only one and the same homosexual partner for at least six months. Moreover, having no anal sexual intercourse in the past 6 months or using condom every time having anal sexual intercourse with homosexual partners was categorized as always using condom in the past 6 months.

#### 2.4.3. Knowledge of HIV

Eight HIV-related questions were administered to evaluate the participants' knowledge of HIV [16]. The questions included the following: could HIV be transmitted via mosquito bites? Do individuals infected with HIV look healthy? Could HIV be transmitted via blood transfusion or using blood products? Could HIV be transmitted via sharing a meal with a person infected with HIV? Could HIV be transmitted to an unborn child via delivery? Could HIV be transmitted via sharing a needle with someone who was infected with HIV? Could the risk of being infected with HIV be reduced by having sex with a steady sexual partner? And, could HIV be transmitted if you correctly use a condom every time you have sex? One point was given to each correct answer and zero point was given to each wrong answer. Thus the total score, which ranged from 0 to 8 points, was equal to the total number of correct answers. Participants who scored a total of at least 6 points were regarded as having a comprehensive knowledge of HIV [17].

#### 2.4.4. HIV and Syphilis Tests

Blood samples were collected from all respondents for HIV and syphilis tests. HIV was tested using enzyme-linked immunosorbent assay (ELISA) (Wantai Biotech Inc, Beijing, China) for screening and the screening results were confirmed using Western blot (MP Diagnostics, Singapore). Syphilis was tested with ELISA (Lizhu Biotech Inc, Zhuhai, China) for screening and then the screening results were confirmed by toluidine red unheated serum test (TRUST) (Rongsheng Biotech Inc, Shanghai, China). Participants with ELISA positive and Western blot positive were diagnosed as HIV positive (HIV infection); otherwise they were diagnosed as HIV negative. Similarly, participants with ELISA positive and TRUST positive were diagnosed as having syphilis infection.

### 2.5. Data Analyses

Statistical analyses were performed using Statistical Package for the Social Science (SPSS) version 19.0 (IBM Corp, Armonk, NY). All statistical tests were two-tailed and were considered significant if *P* < 0.05. For categorical variables, frequency (%) was presented, while mean and standard deviation (SD) were presented to describe a continuous variable. The chi-square (*χ* ^2^) test was used to compare the distribution of sociodemographic and risky sexual variables between HIV positive and HIV negative MSM. Also, it was used to compare the distribution of early sexual debut between HIV positive and HIV negative MSM. The independent sample student *t*-test was used to compare age at first sexual intercourse by sociodemographic characteristics of MSM. Univariable logistic regression analyses were used to assess the effect of early sexual debut on HIV infection status. Multivariable logistic regression analyses were performed to explore the contribution of early sexual debut to HIV infection status adjusting for the covariates including sociodemographic variables, risky sexual variables, syphilis infection status, and knowledge of HIV. Each logistic regression model included only one indicator of early sexual debut so as to avoid multicollinearity.

## 3. Results

### 3.1. Participants' Characteristics

A total of 537 participants were interviewed. After excluding 4 invalid questionnaires, 533 participants were finally enrolled in this study.

Table 1 presents sociodemographic and risky sexual characteristics of the participants. Among all participants, 499 (93.6%) were of Han nationality, 160 (30.0%) had attended junior middle school at most, and 52 (9.8%) were categorized as having socioeconomic disconnection. The mean (SD) present age of the participants was 32.09 (8.41) years.

Furthermore, 354 (66.4%) reported having ever had vaginal sexual intercourse and 368 (69.0%) reported having a fixed homosexual partner. There were 214 (42.5%) participants who reported always using condoms in anal sexual intercourse in the past six months and 378 (70.9%) participants who reported having a comprehensive knowledge of HIV. Those who were confirmed as having syphilis infection were 159 (29.8%) of the participants.

### 3.2. Prevalence of HIV Infection

Prevalence rate of HIV infection among MSM in this study was 24.2% (129/533). The association between HIV infection and sociodemographic variables and the association between HIV infection and risky sexual variables are presented in Table 1. Role in the anal sexual intercourse was significantly associated with HIV infection (*P* < 0.05), and MSM with syphilis infection were more likely to be infected with HIV than those without syphilis infection (44.0% versus 15.8%, *P* < 0.05). Besides, those who always used condom in anal sexual intercourse in the past 6 months were less prone to be infected with HIV, compared with those who did not always use condom in anal sexual intercourse in the past 6 months (18.2% versus 27.7%, *P* < 0.05).

### 3.3. Age at First Sexual Intercourse

Table 2 presents age (mean ± SD) of MSM at first sexual intercourse with respect to sociodemographic variables. The mean (SD) ages at first vaginal sexual intercourse, first anal sexual intercourse, and first sexual intercourse were 21.38 (3.93), 22.43 (6.40), and 19.87 (3.80) years, respectively. Age at first sexual intercourse was significantly associated with some sociodemographic variables, namely, present age, marital status, educational level, and socioeconomic disconnection (*P* < 0.05). Compared with participants with present age of at least 25, participants with present age younger than 25 had earlier age at first sexual intercourse (20.30 versus 17.81, *P* < 0.05), first vaginal sexual intercourse (23.28 versus 18.41, *P* < 0.05), and first anal sexual intercourse (21.85 versus 17.73, *P* < 0.05). In addition, compared with the married individuals, those who were never married/divorced/widowed had earlier age at first sexual intercourse (20.88 versus 19.54, *P* < 0.05), first vaginal sexual intercourse (22.65 versus 20.67, *P* < 0.05), and first anal sexual intercourse (27.29 versus 20.88, *P* < 0.05).

### 3.4. Association between Early Sexual Debut and HIV Infection

Table 3 presents the distribution of early sexual debut by HIV infection status. The proportions of participants reported having experienced sexual debut and anal sexual debut at the age of 14 or less were 5.6% and 4.5%, respectively. In addition, among 354 MSM who had ever had sex with females, 2.0% reported having vaginal sexual debut at the age of 14 or less. Early VSD and early sexual debut were not significantly associated with HIV infection (*P* > 0.05). However, the prevalence of HIV infection was significantly higher among individuals who reported having experienced early ASD than among those who did not experience early ASD (41.7% versus 23.4%, *P* < 0.05).

Logistic regression analyses were performed to examine some factors which might be associated with HIV infection in this group of MSM. The results are shown in Table 4. Univariable logistic regression analyses showed that early ASD (odds ratio (OR) = 2.34, 95% confidence interval (95% CI) = 1.02–5.41) was a risk factor for HIV infection and remained a significant risk factor (OR = 2.88, 95% CI = 1.01–8.18) for HIV infection adjusting for the covariates listed in Table 1. However, both univariable and multivariable logistic regression analyses indicated that early sexual debut and early VSD were not significantly associated with HIV infection status (*P* > 0.5).

## 4. Discussion

Although some studies indicated that early sexual debut was associated with HIV infection [18], only a handful of studies investigated early sexual debut and HIV infection among MSM [7]. Motivated by the fact that some MSM also have vaginal sexual intercourse with women, to the best of our knowledge, this may be the first study in China to classify early sexual debut into early anal sexual debut and early vaginal sexual debut when exploring the association between early sexual debut and HIV infection among the Chinese MSM in China.

The prevalence of HIV infection among MSM in this study population was 24.2%, which is higher than the prevalence, 3.3–5.3%, of HIV infection found among the general MSM in the same area [19–21]. This is mainly due to the fact that the prevalence of STDs among MSM recruited from an STD clinic was much higher than that among the general MSM [19]. Besides, the HIV infection prevalence found in this study was almost 5 times higher than that (4.2%) reported in a previous study which enrolled 743 MSM from the STD clinic of Shenzhen Institute of Dermatology in 2008-2009 [19]. Thus, the prevalence of HIV infection in this study supports the results of a previous study which indicated an alarming increase of HIV infections among MSM in China in recent years [1] and, hence, the suggestions made by this previous study to urgently implement more efficient preventions and more comprehensive interventions of HIV infections among MSM are supported.

Some risky sexual behaviors, including infrequent condom use in anal sexual intercourse in the past 6 months and role in anal sexual intercourse, were correlated with HIV infection. This finding was consistent with previous studies which investigated risk factors for HIV infection [2, 22]. Indeed, promoting condom use in anal sexual behavior is a pivotal approach in order to reduce the risk of HIV infection among MSM [23]. The significant association of syphilis infection with HIV infection in this study was also consistent with previous research findings which established a higher likelihood of HIV infection in someone with syphilis infection than in someone without syphilis infection [24, 25]. However, unlike in some previous researches [17, 22], comprehensive knowledge of HIV in this study was not significantly associated with HIV infection. The reason for the insignificant association could be due to even promotion of HIV knowledge by the local government or communities. Nevertheless, it is widely understood that increasing knowledge about HIV could reduce the prevalence of HIV infection [26].

Similar to a previous study [27], the proportion (66.4%) of the MSM who also have vaginal sexual intercourse with women in this study population was high, suggesting that potential risk factors for HIV infection among MSM should be considered from an HIV transmission web involving sexual intercourse activities among MSM and between MSM and women. It was also found that the mean ages at first vaginal sexual intercourse, first anal sexual intercourse, and first sexual intercourse were 21.38, 22.43, and 19.87 years, respectively. Consistent with a previous study [28], age at first sexual intercourse was significantly related to some sociodemographic characteristics. For example, having a younger present age (<25), being never married/divorced/widowed, having education level at most junior middle school, and having a socioeconomic disconnection were significantly associated with a relatively younger age at first sexual intercourse. Given that a relatively younger age at first sexual intercourse increased the likelihood of being infected with STDs in later life [7], it is necessary to give special concern to the MSM with those preceding associated factors for having first sexual intercourse at a relatively younger age.

Also found in this study is that early ASD was associated with HIV infection, while early VSD was not associated with HIV infection. This can be explained by the fact that compared with male sexual partners of MSM female sexual partners of MSM are usually fixed, and thus male sexual partners of MSM are more likely to transmit HIV or syphilis [29]. For example, it was found that having a male as the first sexual partner rather than a female was a risk factor for syphilis infection [30], and it has been widely understood that early ASD was related to many subsequent risky sexual behaviors, such as more anal sexual partners, more unprotected anal sexual intercourse, and more sex exchange [5, 8]. Thus, MSM with ASD, rather than VSD, are at higher risk for HIV infection.

There may be several ways in which early sexual debut affects one's susceptibility of being infected with HIV. Firstly, early sexual debut may increase one's risk of HIV infection by extending one's duration of sexual activity, since compared with those who do not have an early sexual debut individuals who commence sex early may have a relatively longer duration of sexual activity and therefore they would be potentially exposed to HIV infection for a longer period of time [31, 32]. Secondly, individuals who start sex early may also be more prone to be related to many subsequent risky sexual behaviors, such as having more sexual partners and less condom use [6, 33]. Finally, it is possible that individuals who commence sex early are more likely to have partners with higher risk of being infected with HIV than those who do not have an early sexual debut [5].

This study has some limitations. Firstly, the participants were recruited using convenience sampling method. Though widely used, this method is improper for random sampling, which may reduce the generalizability of our findings to other MSM populations. Secondly, participants in this study were recruited from the STD clinic, as a consequence of which selection bias may be induced. Thirdly, though managed by well-trained investigators, this survey was essentially retrospective, suggesting that some data from questions, such as “how many anal sexual partners did you have in the past 6 months?” and “how old were you at your first sexual intercourse?,” could be approximations which might be affected by recall bias. Finally, the results of this study may not be applicable to the MSM population with a history of drug use because MSM with a history of drug use were excluded from this study.

## 5. Conclusions

This cross-sectional study mainly explored the association between early sexual debut and HIV infection among MSM in Shenzhen, China. The prevalence of HIV infection among this group was 24.2% and 66.4% of the MSM reported having had vaginal sexual intercourse with females. The mean ages at first vaginal sexual intercourse, first anal sexual intercourse, and first sexual intercourse were 21.38, 22.43, and 19.87 years, respectively. Having a younger present age (<25), being never married/divorced/widowed, having attended junior middle school at most, and being socioeconomically disconnected were associated with a relatively younger age at first sexual intercourse. Compared with the MSM who had anal sexual debut at the age of greater than 14 years, those who had anal sexual debut at the age of 14 or less were more prone to be infected with HIV. Therefore, early and effective measures to reduce the transmission of HIV among MSM in Shenzhen should be taken, and MSM with early sexual debut should be given special consideration.

## Figures and Tables

**Table 1:** Sociodemographic and risky sexual characteristics of MSM by HIV infection status.

Variables	Category	Total sample (%)	HIV positive (%)	HIV negative (%)	*χ* ^2^ value	*P* value
Present age					2.141	0.143
	<25	93 (17.4)	28 (30.1)	65 (69.9)		
	≥25	440 (82.6)	101 (23.0)	339 (77.0)		
Ethnicity					0.537	0.464
	Han	499 (93.6)	119 (23.8)	380 (76.2)		
	Minority	34 (6.4)	10 (29.4)	24 (70.6)		
Marital status					2.158	0.142
	Married	129 (24.2)	25 (19.4)	104 (80.6)		
	Never married/divorced/widowed	404 (75.8)	104 (25.7)	300 (74.3)		
Educational level					2.577	0.108
	≤junior middle school	160 (30.0)	46 (28.8)	114 (71.2)		
	>junior middle school	373 (70.0)	83 (22.3)	290 (77.7)		
Socioeconomic disconnection					0.232	0.630
	Yes	52 (9.8)	14 (26.9)	38 (73.1)		
	No	481 (90.2)	115 (23.9)	366 (76.1)		
Monthly income (RMB)					2.460	0.117
	<2500	73 (13.7)	23 (31.5)	50 (68.5)		
	≥2500	460 (86.3)	106 (23.0)	354 (77.0)		
Length of stay in Shenzhen					0.100	0.752
	<1 month	38 (7.1)	10 (26.3)	28 (73.7)		
	≥1 month	495 (92.9)	119 (24.0)	376 (76.0)		
Syphilis infection					48.539	<0.001
	Yes	159 (29.8)	70 (44.0)	89 (56.0)		
	No	374 (70.2)	59 (15.8)	315 (84.2)		
Ever had vaginal sexual intercourse					0.620	0.431
	Yes	354 (66.4)	82 (23.2)	272 (76.8)		
	No	179 (33.6)	47 (26.3)	132 (73.7)		
Fixed homosexual partner					0.054	0.816
	Yes	368 (69.0)	88 (23.9)	280 (76.1)		
	No	165 (31.0)	41 (24.8)	124 (75.2)		
Number of anal sexual partners in the past 6 months					5.285	0.071
	≤2	217 (40.7)	44 (20.3)	173 (79.7)		
	3–5	150 (28.1)	46 (30.7)	104 (69.3)		
	>5	166 (31.2)	39 (23.5)	127 (76.5)		
Role in anal sexual intercourse^*∗*^					44.861	<0.001
	Insertive	212 (41.1)	21 (9.9)	191 (90.1)		
	Receptive	123 (23.8)	50 (40.7)	73 (59.3)		
	Both	181 (35.1)	54 (29.8)	127 (70.2)		
Always use condom in anal sexual intercourse in the past 6 months^*∗*^					6.089	0.014
	Yes	214 (42.5)	39 (18.2)	175 (81.8)		
	No	289 (57.5)	80 (27.7)	209 (72.3)		
With a comprehensive knowledge of HIV					0.114	0.736
	Yes	378 (70.9)	93 (24.6)	285 (75.4)		
	No	155 (29.1)	36 (23.2)	119 (76.8)		

^*∗*^Data may not add up to 533 because of missing value.

**Table 2:** Age (mean ± SD) at first sexual intercourse by sociodemographic characteristics.

Variables	Category	Age at first vaginal sexual intercourse	Age at first anal sexual intercourse	Age at first sexual intercourse
Present age				
	<25	18.41 ± 2.76	17.73 ± 2.32	17.81 ± 2.72
	≥25	23.28 ± 6.63^*∗*^	21.85 ± 3.84^*∗*^	20.30 ± 3.85^*∗*^
Ethnicity				
	Han	21.46 ± 3.90	22.48 ± 6.37	19.92 ± 3.77
	Minority	20.21 ± 4.19	21.76 ± 6.98	19.10 ± 4.21
Marital status				
	Married	22.65 ± 4.11	27.29 ± 8.46	20.88± 3.95
	Never married/divorced/widowed	20.67 ± 3.64^*∗*^	20.88 ± 4.63^*∗*^	19.54 ± 3.70^*∗*^
Educational level				
	≤junior middle school	20.79 ± 3.70	22.65 ± 7.57	19.16 ± 3.34
	>junior middle school	21.66 ± 4.01	22.34 ± 5.84	20.17 ± 3.95^*∗*^
Socioeconomic disconnection				
	Yes	19.97 ± 3.10	21.96 ± 8.17	18.62 ± 2.82
	No	21.51 ± 3.97^*∗*^	22.48 ± 6.19	20.00 ± 3.87^*∗*^
Monthly income (RMB)				
	<2500	22.15 ± 3.86	24.05 ± 9.26	19.33 ± 4.07
	≥2500	21.26 ± 3.93	22.17 ± 5.79^*∗*^	19.95 ± 3.75
Length of stay in Shenzhen				
	<1 month	21.19 ± 3.75	21.58 ± 5.84	19.79 ± 3.43
	≥1 month	21.39 ± 3.94	22.50 ± 6.45	19.88 ± 3.83

Analysis of *t*-test, ^*∗*^ *P* < 0.05.

**Table 3:** Distribution of early sexual debut by HIV infection status.

Variables	Category	Total sample (%)	HIV positive (%)	HIV negative (%)	*χ* ^2^ value	*P* value
Early VSD					0.117	0.732
	Yes	7 (2.0)	2 (28.6)	5 (71.4)		
	No	347 (98.0)	80 (23.1)	267 (76.9)		
Early ASD					4.178	0.041
	Yes	24 (4.5)	10 (41.7)	14 (58.3)		
	No	509 (95.5)	119 (23.4)	390 (76.6)		
Early sexual debut					2.692	0.101
	Yes	30 (5.6)	11 (36.7)	19 (63.3)		
	No	503 (94.4)	118 (23.5)	385 (76.5)		

**Table 4:** Logistic regression analyses for assessing the effect of early sexual debut on HIV infection status.

Variables	Category	Univariable analysis		Multivariable analysis	
		OR (95% CI)	*P* value	OR (95% CI)	*P* value
Early VSD			0.733		0.885
	No	1		1	
	Yes	1.34 (0.25–7.01)		1.15 (0.18–7.57)	
Early ASD			0.046		0.047
	No	1		1	
	Yes	2.34 (1.02–5.41)		2.88 (1.01–8.18)	
Early sexual debut			0.106		0.150
	No	1		1	
	Yes	1.89 (0.87–4.08)		2.00 (0.78–5.11)	
